# A Biomechanical Study of Calcaneal Tuberosity Avulsion Fracture: A Comparison Between Three-Screw Versus Two-Screw Fixation Strength

**DOI:** 10.7759/cureus.56967

**Published:** 2024-03-26

**Authors:** Kwong-Lee Wan, Sugesh Raghavan, YeokPin Chua, Rukmanikanthan Shanmugam, Mohamad Izani Ibrahim

**Affiliations:** 1 Department of Orthopaedics and Traumatology, Raja Permaisuri Bainun Hospital, Ipoh, MYS; 2 Department of Orthopaedic Surgery, National Orthopaedic Centre of Excellence for Research and Learning (NOCERAL) University of Malaya, Kuala Lumpur, MYS; 3 Department of Orthopaedics, Sunway Medical Centre, Subang Jaya, MYS; 4 Department of Orthopaedics, Prince Court Medical Centre, Kuala Lumpur, MYS

**Keywords:** calcaneal achilles tendon pull, calcaneal beak-type fracture, calcaneal tongue-type fracture, calcaneum screw fixation, calcaneal tuberosity fracture, calcaneal avulsion fracture

## Abstract

A calcaneal tuberosity avulsion fracture constitutes a sub-group of calcaneal fractures and it poses a high risk of soft tissue compromise, necessitating urgent reduction and fixation. This fracture was commonly treated with screw fixation, and fixation failure associated with this method has been reported in the literature. In light of this, we tested and compared the strength of two-screw (2S) versus three-screw (3S) fixations, where the third screw was fixed from the posterior calcaneal tuberosity towards the anterior process in addition to the two parallel screws. Synthetic calcaneum models were tested with an Instron machine to measure the maximum tensile load and stiffness. The mean maximum tensile loads for 3S and 2S were 455.8 N (SD = 47.4) and 341.0 N (SD = 30.9), respectively, and the difference was statistically significant. The mean stiffnesses for 3S and 2S were 29.2 N/mm (SD = 1.8) and 29.7 N/mm (SD = 2.0), but this difference did not reach statistical significance. Based on our findings, the added third screw increased the pull-out strength and can be inserted percutaneously to minimize soft tissue compromise.

## Introduction

A calcaneal tuberosity avulsion fracture occurs due to the sudden pull by the Achilles tendon, which causes the upper segment to be avulsed proximally. This displaced bony fragment puts the posterior ankle soft tissue at risk of impingement. Gardner et al. reported that 21% of the 127 cases in their study had soft tissue compromise, which required further procedures, and one case ended up with amputation [1]. Gitajn et al. reported that 39.4% of their cases with soft tissue compromise at presentation went on to experience subsequent soft tissue issues [2]. Delay in managing this sub-group of calcaneal fractures could cause full-thickness soft tissue necrosis [3], leading to an exposed Achilles tendon and/or calcaneum, which may put the limb at risk of amputation. Therefore, urgent reduction and fixation are required to reverse the soft tissue impingement in these patients. However, the reduction and fixation techniques usually need to traverse through this precarious soft tissue area.

Percutaneous screw fixation is a logical choice of fixation to minimize further soft tissue compromise. The associated skills and instruments are familiar to all orthopedic surgeons. However, failures of the screw fixation technique have been reported in case reports, probably due to the significantly higher pulling force by the Achilles tendon [4-7]. Therefore, we compared the biomechanical strengths of three-screw (3S) versus two-screw (2S) fixation, under the hypothesis that there was no significant difference in pull-out strength between the 3S and 2S groups.

## Materials and methods

**Figure 1:**
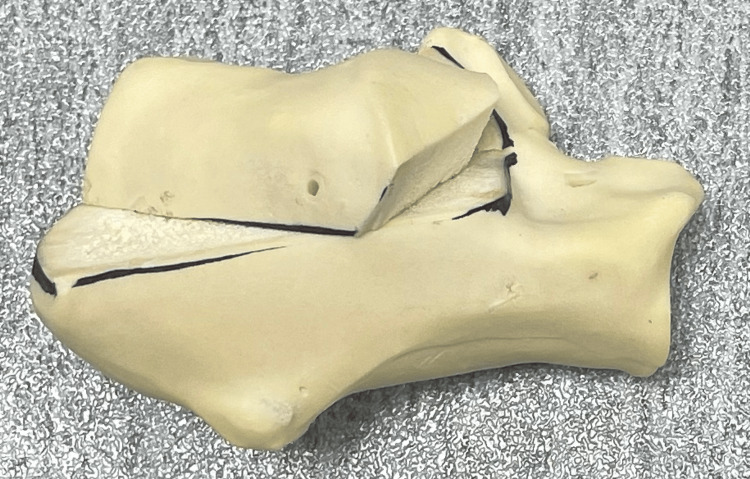
Cut model of the right calcaneum synthetic model The anterior wedge was cut to eliminate the potential blockage when the fragment at the posterior tuberosity was rotated

The right-side calcaneum synthetic model by Synbone (Synbone, Johor, Malaysia; a subsidiary of Synbone AG Switzerland) was used as the testing model. A total of 14 models were produced in the same batch simultaneously. The Essex-Lopresti tongue-type fracture was chosen as the fracture pattern model. The tongue-type calcaneal fracture line and the portion for the chamfer cut for removal were drawn, as shown in Figure 1. The fracture line traversed from the midpoint of the posterior tuberosity towards the inferior end of the posterior facet to simulate Sanders IIC fracture. The fracture line started at 5 mm proximal to the protuberance of the posterior tuberosity and cut towards the inferior edge of the posterior facet. The fracture was created with an oscillating saw. The chamfer cut with the wedge removed at the anterior portion was created to minimize blockage when the fragment rotated proximally as pulled by the rope, simulating the Achilles tendon pull. All models were cut and randomized into two groups with seven samples for each group (n = 7).

**Figure 2:**
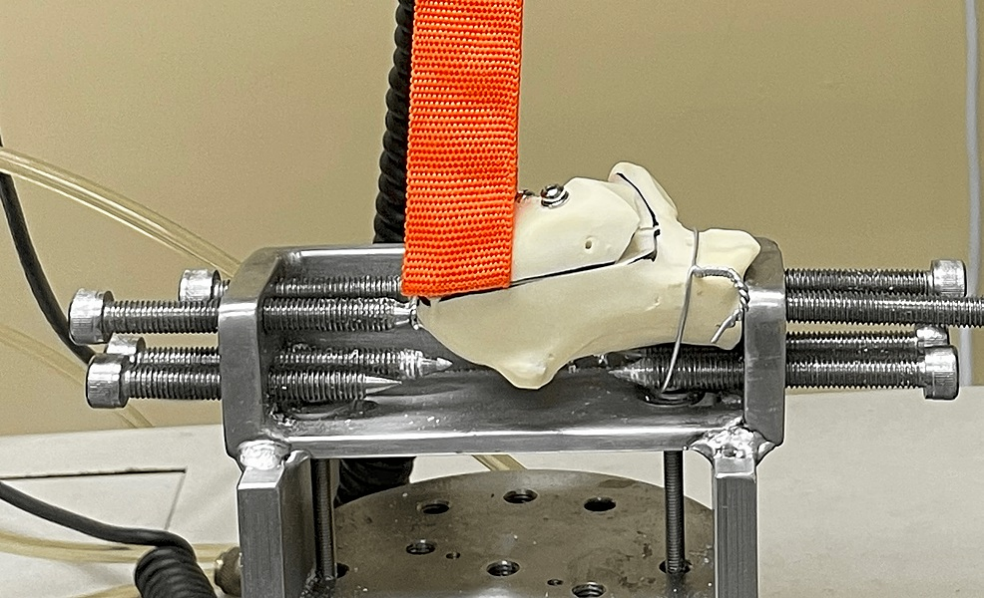
Mounted right-side calcaneum synthetic model The mounting posterior pin was fixed into the 6.5 mm screw that was embedded into the model. The mounting anterior pin was fixed into the anterior process and augmented with a cerclage wire to minimize rotation motion. A third mounting pin was fixed to the medial wall to reduce the yaw axis motion. The flat rope was ‘sandwiched’ when the screws were fixed and compressed the fracture gap

Each model was fixed to the base plate at the anterior process and augmented with cerclage wire, as shown in Figure 2. A cannulated screw with a diameter of 6.5 mm and a length of 35 mm was inserted into the posterior-inferior part of the tuberosity, which was used as a solid point of fixation. This point of fixation was 5 mm inferior to the fracture line. A third point of fixation was fixed from the lateral side of the calcaneum, and this third point of fixation and the cerclage wire of the anterior process minimized rotation instability.

**Figure 3:**
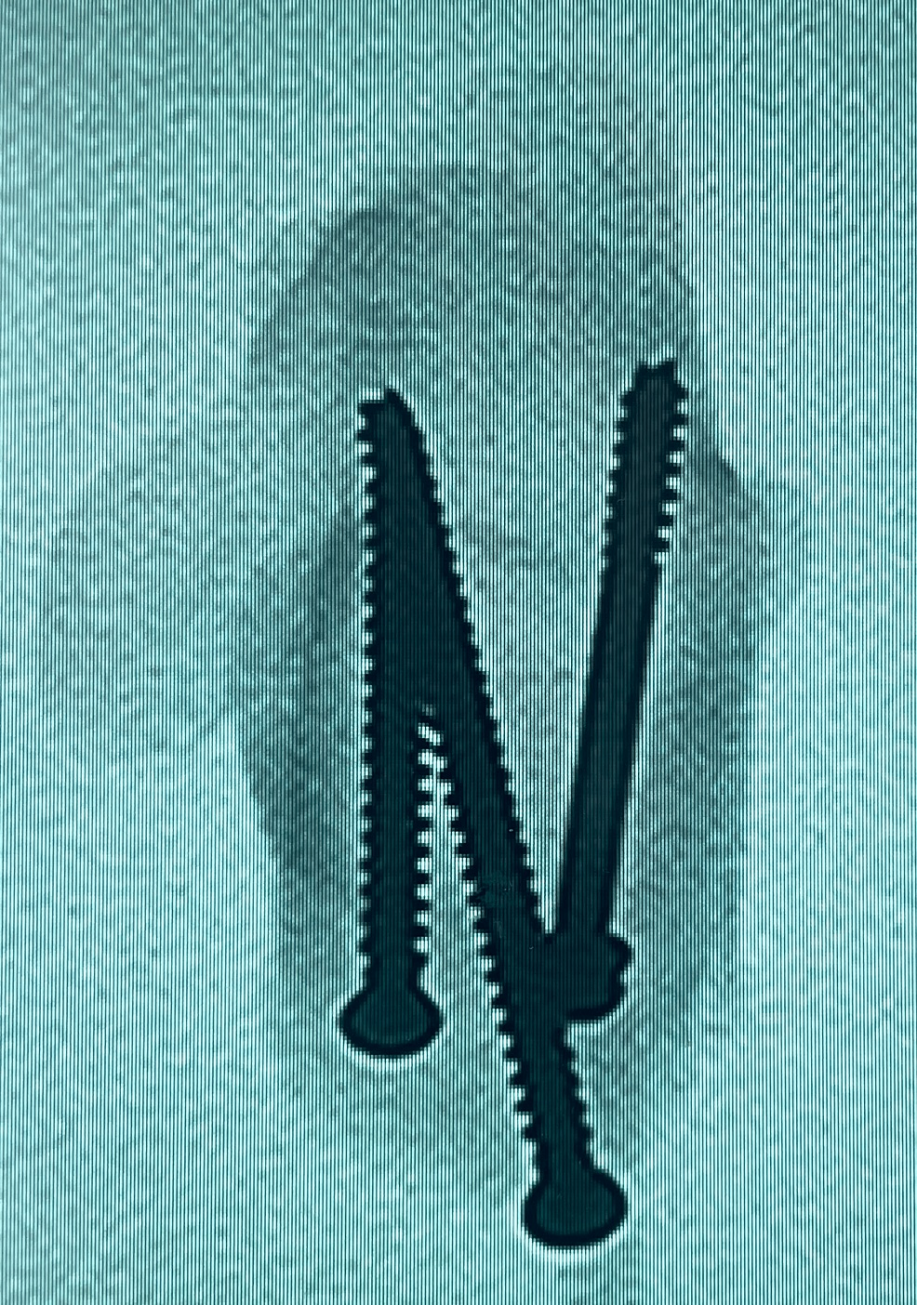
Radiograph axial view of the calcaneum - three-screws (3S) construct trajectories

**Figure 4:**
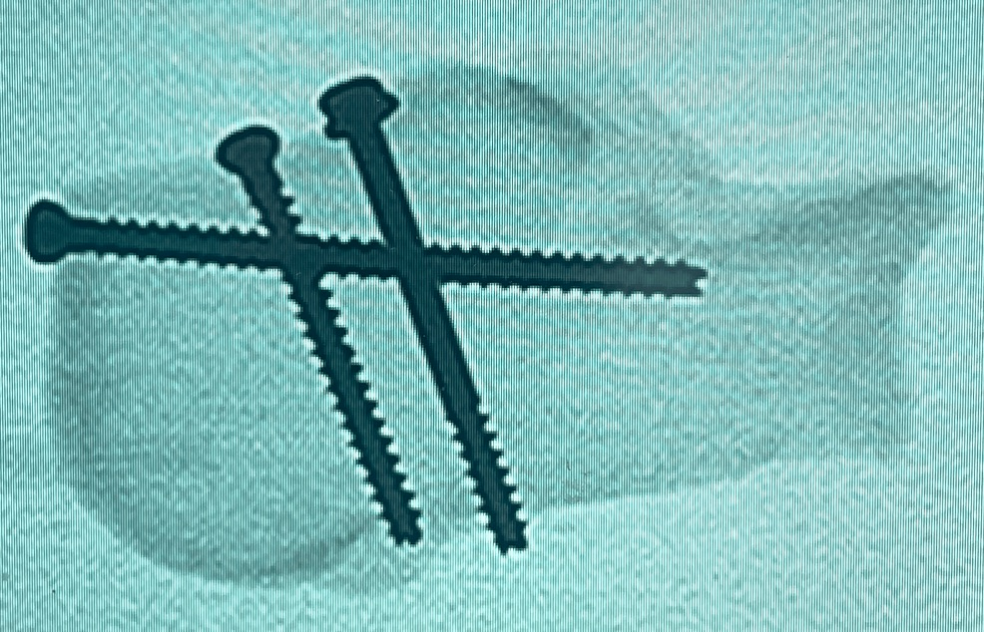
Radiograph lateral view of the calcaneum - three-screws (3S) construct trajectories

**Figure 5:**
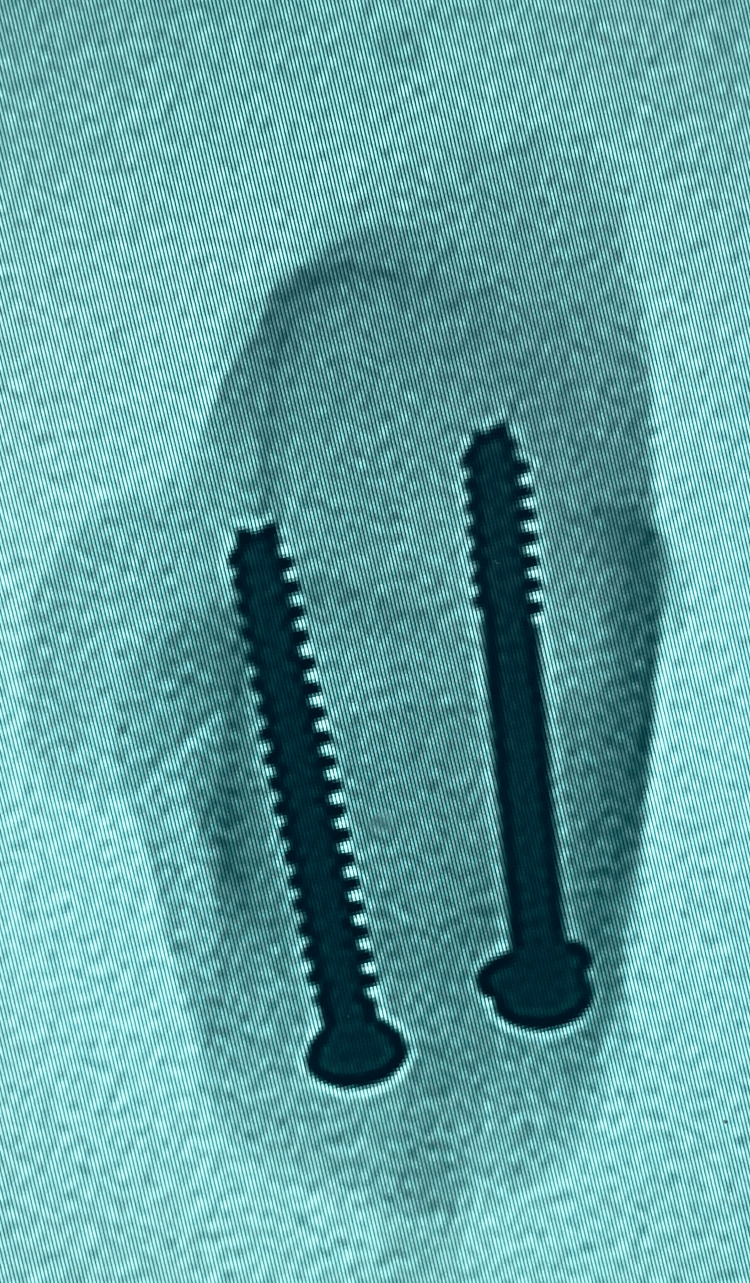
Radiograph axial view of the calcaneum - two-screws (2S) construct trajectories

**Figure 6:**
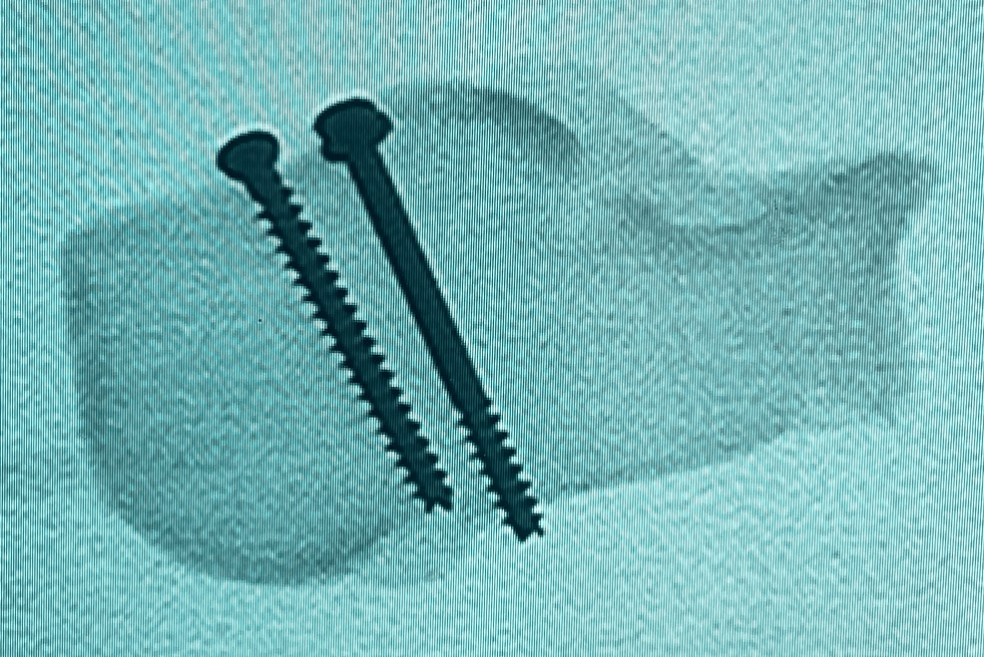
Radiograph axial view of the calcaneum - two-screws (2S) construct trajectories

The flat rope was applied between the fracture fragments before fixation, as shown in Figure 2 after mounting the model. The flat rope was intended to mimic the Achilles tendon pull without causing any potential weakness to the model. The rope was ‘sandwiched’ upon fracture fixation. The fracture was fixed accordingly to three screws construct (3S) and two screws construct (2S), as shown in Figures 3-6. All screws were made of stainless steel and were medical-grade screws for clinical use (Hardik International, Rajkot, India). In the 3S group, a half-threaded cannulated cancellous screw with a diameter of 4.0 mm and a length of 45 mm with a washer was inserted at first at the lateral half of the calcaneum tuberosity perpendicular to the fracture line for compression effect. The 45 mm length of this first screw was determined by the depth of the lateral half of the calcaneum. The second screw was a fully threaded cannulated cancellous screw with a diameter of 4.0 mm and a length of 40 mm that was inserted at the medial half of the calcaneum parallel to the first screw and perpendicular to the fracture line for the anti-rotation effect. The 40 mm length of this second screw was determined by the shorter depth of the medial half of the calcaneum. A third screw, which was a fully threaded cannulated cancellous screw with a diameter of 4.0 mm and a length of 60 mm, was inserted from the posterior-superior tuberosity, directed towards the anterior process, and traversed between the initial two screws. In the 2S group, two screws were inserted similar to the first two screws in the 3S group, and the third long screw was omitted. All screws were inserted by a single surgeon. Guidewires were placed through and through to ensure all trajectories were consistent before drilling and inserting the screws.

The load cell used had a 2000 N capacity. The Instron® 3365 tabletop universal testing series with Bluehill® software was the testing machine used in extension mode. The pre-test load was set at 40 N. Pre-test cycling was set at 1 mm/second speed, 1 mm distance, and cycled 10 times. Pre-test cycling is a practice used to minimize slacks in mounting instruments, as demonstrated in the study by Wicks et al. [8]. The actual test was set at 0.5 mm/second speed and a distance of 20 mm. The end of the test was set at 1000 N with the intention of testing to failure or a fracture gap of 5 mm. The 1000 N limit was chosen because other biomechanic studies did not exceed 1000 N of force [9-11].

The tensile load to cause the 5 mm fracture gap was measured in Newtons (N), and the stiffness was measured in Newtons per millimeter (N/mm). Data normality was tested with the Shapiro-Wilk test, and a p-value above 0.05 was set to show a normal data distribution. The independent t-test was used for means comparison if the data showed a normal data distribution and a p-value below 0.05 was set to show a statistically significant comparison. The statistical analysis was performed with SPSS Statistics software Version 26.0 (IBM Corp., Armonk, NY).

This study received approval from the National Medical Research Registry (NMRR), Malaysia. The research ID was RSCH ID-23-03408-IMB, and the NMRR ID was NMRR ID-23-02325-EJV.

## Results

There were seven samples (n = 7) in the 3S group. The tensile load and stiffness data at the fracture gap of 5 mm had normal distribution data as tested with the Shapiro-Wilk test. The means and standard deviations of maximum load and stiffness were 455.8 N (SD = 47.4) and 29.2 N/mm (SD = 1.8), respectively (Table 1).

**Table 1:** The mean tensile load and stiffness that caused a 5 mm fracture gap for three-screws (3S) construct Both tensile load and stiffness data were normally distributed as tested with the Shapiro-Wilk test (p>0.05) SD: standard deviation

Three-screws (3S) construct		
Sample number	Tensile load (N)	Stiffness (N/mm)
1	413.2	27.7
2	437.8	25.8
3	472.8	30.5
4	454.1	29.8
5	427.6	29.6
6	554.0	30.0
7	431.1	30.7
Shapiro-Wilk test p-value	0.055	0.078
Mean (SD)	455.8 (47.4)	29.2 (1.8)

There were seven samples (n = 7) in the 2S group. The tensile load and stiffness data at the fracture gap of 5 mm had normal distribution data as tested with the Shapiro-Wilk test. The mean and standard deviations of maximum load and stiffness were 341.0 N (SD = 30.9) and 29.7 N/mm (SD = 2.0), respectively (Table 2).

**Table 2:** The mean tensile load and stiffness that caused a 5 mm fracture gap for two-screws (2S) construct Both tensile load and stiffness data were normally distributed as tested with the Shapiro-Wilk test (p>0.05) SD: standard deviation

Two-screws (2S) construct		
Sample number	Tensile load (N)	Stiffness (N/mm)
1	348.0	30.4
2	377.1	31.7
3	314.6	30.1
4	311.7	25.7
5	316.7	30.3
6	331.4	28.6
7	387.2	31.0
Shapiro-Wilk test p-value	0.170	0.123
Mean (SD)	341.0 (30.9)	29.7 (2.0)

The comparison of the mean tensile load between 2S and 3S was statistically significant, with a p-value of 0.0003. The mean 3S tensile load was higher than the mean 2S tensile load by 114.8 N (95% confidence interval: 68.3, 161.4). The comparison of the mean stiffness between 2S and 3S was not statistically significant, with a p-value of 0.6295, which was above 0.05 (Table 3).

**Table 3:** Comparison of the mean tensile load and stiffness using independent t-test The difference in mean tensile load was statistically significant (p<0.05). The difference in mean stiffness was not statistically significant (p>0.05) SD: standard deviation; CI: confidence interval

Parameters	Mean (SD)		Mean difference (95% CI)	P-value
	Three-screws (3S) construct (n = 7)	Two-screws (2S) construct (n = 7)		
Tensile load (N)	455.8 (47.4)	341.0 (30.9)	114.8 (68.3, 161.4)	0.0003
Stiffness (N/mm)	29.2 (1.8)	29.7 (2.0)	0.5 (1.7, 2.7)	0.6295

## Discussion

The calcaneum tuberosity fracture can be considered an avulsion fracture, and it has been classified into types I, II, and III by Beavies et al. [12]. Type I consists of a small fragment avulsed from the Achilles tendon insertion. Type II is a major avulsion bony fracture from the tuberosity extending towards the posterior facet, which was described as having a ‘beak’ appearance in the lateral view radiograph. Type III involves an insertional avulsion of the Achilles tendon inferior to the bursa. An axial force on the calcaneum can present a secondary fracture line in the form of a tongue-type fracture, as described by Essex-Lopresti [13]. Both of these calcaneal fractures are attributed to the Achilles tendon pull. Beavis type II and Essex-Lopresti tongue-type calcaneal tuberosity fractures were considered relevant to our study due to the bigger bony fragment for screw fixation.

Although the screw-only fixation technique appeared to cause the least soft tissue damage in the compromised area, failures associated with this technique had been reported [4-7]. Other fixation techniques that we found in the literature, mainly case reports and case series, are described here. The external fixation technique is commonly used for most at-risk skeletal stabilizations. Frame fixation techniques have also been described, especially in cases where the soft tissue is severely compromised and in open fractures. Percutaneous reduction and pinning, and maintenance of ligamentotaxis reduction with a dual-plane external fixator technique are discussed by Dayton et al. [14]. The circular frame technique has been described for open fracture cases, and the pre-bent wire tensioning technique has been used to reduce the tongue-type displacement [15,16]. Some centers may not have the resources and skills for frame fixation.

Data on internal implants to balance the risk of soft tissue compromise and fixation strength are limited in the literature. In 1952, Essex-Lopresti described the steps in reducing and pinning the tongue-type fracture with cast incorporation [13]. The reduction and pinning technique is still relevant. Devendrappa et al. reported no pinsite infection and 80% favorable results among their 20 patients [17]. Tornetta described a similar reduction technique, but fixation was done with two cannulated screws [18]. In that study, 50% had excellent scores, 35% had good scores, 15% had fair scores, and none had a poor score among the 46 patients who were followed up for 3.4 years [18].

Supplementation with wires and sutures has been reported to be successful for the minimal internal implant technique. Cannulated screw fixation with the titanium wiring technique was reported to be successful in three elderly patients [19]. Similar cannulated screw fixation with the cerclage wire technique was reported to be successful in four patients, and the authors suggested that a bigger screw diameter of 7.0 mm could be used for bigger fragments or a screw diameter of 3.5 mm was preferred for smaller fragments [20]. The Tightrope® technique was reported for a calcaneal sleeve fracture, and this technique could be used for tongue-type fractures as well [21]. A distal femur intercondylar bolt that had washers at both ends was used to revise a failed 3-screw fixation in an elderly patient [5]. These techniques with augmentation could require more soft tissue dissection in an area that would be already precarious, as well as specialized instruments and skill sets.

We considered the Achilles tendon pull to be the most significant force to cause failure of fixation for this study, although, in actual trauma, Achilles tendon pull is not the sole cause of the failure. An Achilles tendon can be subjected to up to 3.95 times of body weight force during walking, and the force can be increased up to 7.71 times of body weight during running [22]. In a non-weight-bearing position, ankle plantar flexion can generate 200 N of force at the Achilles tendon; bilateral legs standing ankle plantar flexion can generate 1251 N; and the force can increase to 2760 N in a single-leg stance ankle plantar flexion [23]. Both 3S and 2S fixation strengths were significantly weaker than potential forces, as described above. Thus, compliance with postoperative immobilization and non-weight-bearing ambulation must be adhered to until sufficient union occurs.

Biomechanical studies of calcaneal sub-group fracture fixation are scarce in the literature. A biomechanical study on cadaveric models reported that the 2S construct withstood 280 N force, while the 3-screw construct withstood 470 N force [9]. Khazen et al. have described a 2S construct that withstood 251 N force, and the added anchor suture augmentation increased the strength to 441 N in their biomechanical cadaveric study [10]. In a sawbone biomechanical study, Bacaksiz et al. compared a 2-parallel screw construct, a 2-divergent screw construct, a 2-parallel with one sustentaculum screw, and a 3-parallel screw construct, and the strengths were 606 N, 629 N, 618 N, and 840 N respectively, for a 5 mm gap displacement [11]. The single-stance heel raise, which was reported to load the Achilles tendon up to 2760 N, was far greater than the fixation strengths from the above-mentioned biomechanical studies [23]. Thus, weight-bearing needs to be delayed until full union occurs, while unloaded ankle range of motion can be allowed with screw fixation constructs. No quantitative biomechanic study on other fixation techniques has been published in the literature so far.

We chose this fixation method because the cannulated screw fixation technique could be performed percutaneously, as this injury is commonly associated with compromised soft tissue. Cannulated screws with a diameter of 4.0 mm were chosen because, in some cases, the bony fragments might not be large enough for bigger screws, although bigger screws have a stronger pull-out strength. In the 3S construct, the first screw was a half-threaded screw with a washer for maximal compression. The second screw was a fully-threaded screw for anti-rotation control and to increase the pull-out strength. Both the first and second screws were directed perpendicular to the fracture line to aim for primary bone healing. The third screw was inserted from the posterior tuberosity towards the subthalamic portion of the anterior process of the calcaneum and between the initial two screws. The initial two screws were inserted first for fracture site compression before the third screw was inserted to increase the pull-out strength. This third screw trajectory purchased the denser part of the subthalamic portion of the calcaneum. This screw trajectory has been reported in case reports and a biomechanical cadaveric study [9,18,24].

In our study, the third screw in the 3S group increased the pull-out strength by 114.8 N compared to the 2S group. The 3S average maximum tensile load at 455.8 N indicated that the fixation strength doubled the estimated non-weight-bearing Achilles tendon force at 200 N [23]. This third screw insertion in 3S is a simple additional fixation, which could enable earlier ankle joint mobilization to reduce the risk of ankle stiffness. However, non-weight-bearing status should be maintained, as a bilateral heel-raising stance could generate 1251 N of force at the Achilles tendon, which is significantly higher than the 3S fixation strength [23].

Study strengths

The main strength of our study was the normal distribution of the data, which allowed a parametric test for consistent comparison. The data standard deviations were narrow, indicating a consistent property of the synthetic model. A cadaveric study by Hong et al. showed failure regarding 3-screw and 2S constructs with a 4.1 mm gap at 499.4 N and 315.9 N, respectively [9]. The standard deviations were wider, indicating a possible difficulty in getting more consistent bone density samples [9]. Synthetic bone studies showed narrower standard deviations in the studies by Khazen et al. and Bacaksiz et al. [10,11].

Study limitations

The weakness of this study mainly pertains to the non-anatomical attachment of the simulated Achilles tendon pull. Other types of Achilles tendon attachments created a weakness point at the bone and a wire cut through the bone. Hence, the ‘sandwiched’ rope method was chosen to minimize any potential weakness to the mounting of the models. This study also assumed the Achilles tendon pull to be the only deforming force, when, in an actual trauma, there could be other forces involved. Another weakness was that the fixation was not performed with image intensifier guidance, as it would require a radiation-safe laboratory facility. However, fixation consistency was performed by a single surgeon, and guide-wire entries and exits were matched as closely as possible. A larger sample size would yield better statistical analysis, but it was not possible as the manufacturing of the models was done in a single batch to maintain consistency. Lastly, a bigger-diameter screw such as 6.5 mm could be used as the third screw, but there was concern about the amount of bone stock and a significant weakening of the construct when the bigger screw collided with the initial two smaller screws, causing unreliable sample fixation.

## Conclusions

Although 2S fixation seems adequate to achieve adequate fracture compression, its failure due to the Achilles tendon pull has been reported in the literature. The additional third screw, which was directed toward the subthalamic portion of the calcaneum, was shown in our study to increase the pull-out strength by an additional 114.8 N (95% CI: 68.3, 161.4). This screw could be inserted percutaneously to minimize soft tissue compromise. However, this 3S fixation did not provide sufficient strength for earlier weight-bearing, which needed to be delayed until union occurred.
